# AGAP2-AS1 as a prognostic biomarker in low-risk clear cell renal cell carcinoma patients with progressing disease

**DOI:** 10.1186/s12935-021-02395-9

**Published:** 2021-12-20

**Authors:** Sigrid Nakken, Øystein Eikrem, Hans-Peter Marti, Christian Beisland, Leif Bostad, Andreas Scherer, Arnar Flatberg, Vidar Beisvag, Eleni Skandalou, Jessica Furriol, Philipp Strauss

**Affiliations:** 1 Department of Clinical Medicine, University of Bergen, 5021 Bergen, Norway; 2 Department of Medicine, Haukeland University Hospital, 5021 Bergen, Norway; 3 Department of Urology, Haukeland University Hospital, 5021 Bergen, Norway; 4 Department of Pathology, Haukeland University Hospital, 5021 Bergen, Norway; 5 Spheromics, 81100 Kontiolahti, Finland; 6 Institute for Molecular Medicine Finland (FIMM), University of Helsinki, 00014 Helsinki, Finland; 7 Department of Clinical and Molecular Medicine, Norwegian University of Science and Technology, 7491 Trondheim, Norway; 8 St. Olav’s University Hospital, 7006 Trondheim, Norway

**Keywords:** Biomarker, AGAP2-AS1, ccRCC, Low-risk, RNA-seq

## Abstract

**Background:**

Clear cell renal cell carcinoma (ccRCC) is the most common subtype of renal cancer and one of the most common cancers. While survival for localized ccRCC is good, the survival of metastatic disease is not, and thirty percent of patients with ccRCC develop metastases during follow-up. Although current scoring methods accurately identify patients at low progression risk, a small subgroup of those patients still experience metastasis. We therefore aimed to identify ccRCC progression biomarkers in “low-risk” patients who were potentially eligible for adjuvant treatments or more intensive follow-up.

**Methods:**

We assembled a cohort of ccRCC patients (n  = 443) and identified all “low-risk” patients who later developed progressing tumors (n  = 8). Subsequently, we performed genome-wide expression profiling from formalin-fixed samples obtained at initial surgery from these “low-risk” patients and 16 matched patients not progressing to recurrence with metastasis. The patients were matched for Leibovich sore, creatinine, age, sex, tumor size and tumor stage. Key results were confirmed with qPCR and external data from The Cancer Genome Atlas.

**Results:**

Principal component analysis indicated that systematic transcriptomic differences were already detectable at the time of initial surgery. One thousand one hundred sixty-seven genes, mainly associated with cancer and immune-related pathways, were differentially expressed between progressors and nonprogressors. A search for a classifier revealed that overexpression of *AGAP2-AS1*, an antisense long noncoding RNA, correctly classified 23 of 24 samples, years (4.5 years average) in advance of the discovery of metastasis and without requiring larger gene panels. Subsequently, we confirmed *AGAP2-AS1* gene overexpression by qPCR in the same samples (*p*  < 0.05). Additionally, in external data from The Cancer Genome Atlas, overexpression of *AGAP2-AS1* is correlated with overall unfavorable survival outcome in ccRCC*,* irrespective of other prognostic predictors (*p*  = 2.44E−7).

**Conclusion:**

*AGAP2-AS1* may represent a novel biomarker identifying high-risk ccRCC patients currently classified as “low risk” at the time of surgery.

**Supplementary Information:**

The online version contains supplementary material available at 10.1186/s12935-021-02395-9.

## Introduction

The incidence of kidney cancer is rising worldwide, especially in Western countries [1–3].

Clear cell renal cell carcinoma (ccRCC), the most common subtype of renal cancer, is characterized by an especially poor prognosis [1]. While the 5-year overall survival rate for patients with localized disease is 93% [4], those with metastatic ccRCC have a 5-year survival of only 12% [4]. Notably, however, approximately 30% of patients with localized disease also develop distant metastases during follow-up [5, 6].

Surgery still represents the only curative option for patients with ccRCC [7], but innovative medical therapies are rapidly emerging [1, 5]. Since treatment effectiveness is contingent upon early discovery of the disease or its recurrence, it is critically important to accurately predict the risk of progression, to determine the frequency and type of follow-up, and to increase the chances of timely and successful therapy [5].

A commonly used tool to predict ccRCC recurrence is the Leibovich score, which takes advantage of histological and clinical data to profile progression risk in individual cases [8, 9]. Although this score correctly predicts the 5-year metastasis-free survival rate in 97% of low-risk cases [8], contrary to prediction, a small number of apparently low-risk patients still develop progressive disease. Other assessment methods, also based on clinical and histological data, have similarly been shown to fail to reliably predict disease progression in sizeable groups of low-risk patients [10].

Although these patients, classified as low-risk but developing progressive disease [11], account for  < 5% of low-risk ccRCC patients [8, 12], assuming a broadly similar risk distribution among all European patients, they still represent an approximate annual number of 1500 patients. Failure to identify them prevents their further stratification into subtypes and the timely administration of potentially effective treatments. Once low-risk progressors have been singled out, stratification could also allow for a reduced follow-up for low-risk nonprogressors, freeing valuable resources.

Although initial attempts to characterize low-risk progressors have been made, these results require further validation [12, 13].

In this context, the objective of our study was to identify prognostic biomarkers of potential clinical relevance for predicting ccRCC recurrence in apparently low-risk patients. To address this issue, we took advantage of next-generation sequencing of tumors from a cohort of patients with progressing tumors, despite a low-risk classification according to the Leibovich score, and from matched nonprogressors.

## Patients and methods

### Study design

This retrospective study was planned following REMARK biomarker-research guidelines [14]. As required by REMARK guidelines, it also has to be disclosed that in addition to the experiments described below, RNA sequencing from serum of all participants was also attempted but failed due to RNA fragmentation.

The Regional Ethics Committee (REC) of Western Norway approved the study (REC no. 78-05), and permission for their inclusion was obtained from all participants.

### Patients

Tumor tissues were collected from a cohort of 443 ccRCC patients from Haukeland University Hospital (Bergen, Norway). Each sample was initially examined and scored by an experienced renal pathologist according to Fuhrmann grade. Prior to inclusion in this study each patient was subsequently reassessed and rescored, also by an experienced renal pathologist. The second scoring was performed independently of the first score.

The inclusion criteria were low-risk ccRCC assessed by the Leibovich score (between 0 and 2 according to the 2003 version of the score) [8, 15, 16] and available follow-up data of progression (later occurrence of metastases) or nonprogression (absence of tumor recurrence/metastases); see Additional file 1. Due to an updated Leibovich score being made public, the selected cases were rescored using the updated algorithm [9]. No sample lost its status as low-risk in the new score.

We selected progressors (n  = 8) and included two matched nonprogressors with similar Leibovich scores, Fuhrmann grades, tumor stages and sizes, similar creatinine levels, and similarly underwent surgical tumor removal per progressor sample as controls (n  = 16). Patients who were not treatment naïve, had lymph node metastasis, suffered from heart failure (grade  ≥ 3 according to the New York Heart Association Classification), used immunosuppressive drugs due to transplantation or suffered from severe rheumatic disease at the time of the biopsy were excluded from the study. All patients showed an estimated glomerular filtration rate (eGFR)  > 45 ml/min/1.73 m^2^ and a Charlson comorbidity index (CCI)  > 1, except for one progressor with an eGFR of 36 ml/min and a CCI of 3. See Table 1 for patient details.

**Table 1:** Clinicopathological characteristics of all patients

Unique ID	Age (year)	Gender	Nephrectomy type	Creatinine	Primary tumor status	Tumor size (mm)	Fuhrmann grade	Leibovich score 2003^a^	Leibovich score 2018^b^	Time to metastasis (days)	Pair ID
Rcc9	51	Female	Radical	58	T1a	38	3	2	5	1544	1
Rcc8	63	Male	Radical	60	T1a	40	3	1	3	1994	2
Rcc3	66	Male	Radical	113	T1a	35	3	1	3	2632	3
Rcc11	66	Male	Partial	61	T1a	15	3	2	5	965	4
Rcc1	72	Female	Radical	106	T1a	20	2	0	2	2680	5
Rcc2	72	Male	Radical	109	T1b	50	2	2	5	2319	6
Rcc5	83	Male	Radical	81	T1b	50	2	2	5	109	7
Rcc6	67	Male	Radical	176	T1b	48	2	2	5	1385	8
Rcc13	34	Male	Partial	73	T1a	23	3	1	3		1
Rcc24	47	Female	Partial	48	T1a	40	3	1	3		1
Rcc17	54	Male	Partial	68	T1a	35	3	1	3		2
Rcc20	66	Male	Radical	67	T1a	30	3	1	3		2
Rcc21	57	Male	Partial	73	T1a	30	3	1	3		3
Rcc16	62	Male	Radical	82	T1a	30	3	1	3		3
Rcc23	74	Male	Partial	81	T1a	16	3	1	3		4
Rcc22	66	Male	Partial	83	T1a	38	3	1	3		4
Rcc10	78	Female	Partial	64	T1a	20	2	0	2		5
Rcc18	68	Female	Partial	45	T1a	20	2	0	2		5
Rcc14	72	Male	Partial	97	T1b	55	2	2	5		6
Rcc4	63	Male	Radical	82	T1b	50	2	2	5		6
Rcc7	76	Male	Radical	98	T1b	45	2	2	5		7
Rcc15	75	Male	Radical	73	T1b	45	2	2	5		7
Rcc12	63	Male	Radical	80	T1b	45	2	2	5		8
Rcc19	68	Male	Radical	68	T1b	45	2	2	5		8

Clinicopathological characteristics of progressor (n  = 8) and nonprogressor (n  = 16) patients. Nonprogressors were matched 2:1 to progressors for sex, age, primary tumor stage and size, Fuhrman grade, and eGFR. Pair-ID refers to matched nonprogressors and progressors. No patients exhibited tumor thrombus, sarcomatoid differentiation, or constitutional symptoms. Patients with distant metastases that were not treatment naïve, had lymph node metastasis, suffered from heart failure (grade  ≥ 3 according to the New York Heart Association Classification), or used immunosuppressive drugs due to transplantation or severe rheumatic disease at the time of the biopsy were excluded from the study

^a^Scoring was performed according to the 2003

^b^Scoring was performed according to the 2018 Score

Serum was available from progressors (*n*  = 2) and nonprogressors (*n*  = 6). We also obtained biopsies from the metastasis of 6/8 progressor patients; data not shown.

### Tumor specimens and serum collection

Tissues from all 24 ccRCC patients were stored as formalin-fixed and paraffin-embedded (FFPE) samples at room temperature. Serum was harvested from patient blood samples within 1 h after sampling and subsequently stored at − 80 °C.

### RNA extraction

Four 10 µm sections were cut from the FFPE blocks and used as input, whereas for serum samples, 200 µl was used as input. Total RNA for sequencing and qPCR was extracted, as previously described [17, 18], using the miRNeasy FFPE kit (cat no. 217504; Qiagen, Venlo, The Netherlands). RNA was extracted from serum with the miRNeasy serum/plasma kit (cat no. 217184; Qiagen) according to the manufacturer’s instructions. Following RNA extraction, samples were stored at  − 80 °C.

### RNA yield and gene expression analysis

Total RNA concentration was measured using a Qbit RNA HS assay kit on a Qubit 2.0 fluorimeter (Thermo Fisher Scientific, Waltham, MA, USA). Integrity was assessed using an Agilent RNA 6000 Nano kit on a 2100 bioanalyzer instrument (Agilent Technologies, Santa Clara, CA, USA), and DV200 values were calculated.

RNA extraction from tissue specimens yielded an average of 1362 ng/sample, whereas RNA extraction from serum samples yielded an average of 97 ng/sample.

### RNA library preparation and sequencing

Sequencing libraries were generated using the TruSeq RNA exome library kit (Illumina, San Diego, CA, USA) according to the manufacturer’s instructions.

Libraries were quantitated by qPCR using the KAPA library quantification kit–Illumina/ABI Prism (Kapa Biosystems, Wilmington, MA, USA) and validated using the Agilent high-sensitivity DNA kit on a bioanalyser. Libraries were normalized to 2.6 pM and subjected to cluster and paired-end read sequencing, performed for 2 × 75 cycles on two NextSeq500 HO flow cells (Illumina), according to the manufacturer’s instructions. Sequencing depth was 30 million reads/sample. Base calling was performed using the NextSeq500 instrument and RTA 2.4.6. FASTQ files were generated using bcl2fastq2 conversion software (v.2.17; Illumina).

### Bioinformatics

TopHat (https://ccb.jhu.edu/software/tophat/index.shtml) and Bowtie (http://bowtie-bio.sourceforge.net/index.shtml) were used for assembly of reads and alignment of the contigs to the human genome assembly (GRCh38), respectively. An empirical expression filter was applied, which left genes with  > 1 count per million in at least three samples. Trimmed mean of M values [19] normalization was applied to adjust for variation in library size. Group was used to determine the difference between the two patient groups, and age matching was accounted for as a blocking factor, with one progressor and two nonprogressor samples per age-matched block. Group is here defined as diagnosis, whereas those three age-matched patients constitute a “block” which is factored into the analysis to account for their age-matching.

Comparative analysis was performed using the voom/Limma R package (www.Bioconductor.org) [20, 21]. To reduce unwanted variation induced by unknown sources but avoid overfitting, two surrogate variables were added using the SVA package in R Bioconductor (https://bioconductor.org/packages/release/bioc/html/sva.html).

Genes with a p  ≤ 0.05 and an absolute fold change (abs. FC)  ≥ 2 were considered differentially expressed. Pathway analysis was performed with Ingenuity Pathway Analysis (v.47547484; Qiagen, Redwood City, CA, USA), with the Ingenuity Knowledge Base used as the reference set. Canonical pathways were sorted by the smallest Benjamini–Hochberg adjusted *p* value. Biomarker analysis was performed with the KNN validation package in GenePattern (http://www.broadinstitute.org/cancer/software/genepattern). Euclidean distance was used as the distance measure, where three neighbors were considered, and leave-one-out internal cross-validation was applied. PCA, hierarchical clustering with Ward’s method, and other data visualization techniques were undertaken using JMP Genomics (v.9.0; SAS Institute, Cary, NC, USA) and GraphPad Prism software (v.9.0; GraphPad Software, La Jolla, CA, USA).

#### Gene set enrichment analysis

Gene set enrichment analysis (GSEA) was performed with GSEAv4 (http://www.gsea-msigdb.org/gsea/index.jsp). Normalized gene expression values and their patient group information with the Human Ensembl Gene ID MSigDB 7.4 were tested for enrichment using the KEGG pathway database with 1000-fold permutation of phenotypes, weighted enrichment statistics and signal-to-noise metric for ranking of genes. Gene sets smaller than 15 and larger than 500 were excluded.

### Immunohistochemistry

Immunohistochemistry (IHC) was performed on 4-μm-thick FFPE sections with the following primary antibodies: anti-AGAP2 (1:100; polyclonal, rabbit, no. HPA023474; Sigma–Aldrich, St. Louis, MO, USA) and anti-USP10 (1:1000; monoclonal, rabbit, no. ab109219; Abcam, Cambridge, UK, USA). We included a negative control by including a duplicate of another section and omitting the primary antibody. Incubations were performed overnight at 4 °C and pH 6.0 for both antibodies. Sections were counterstained with hematoxylin (no. CS70030-2; Dako, Kyoto, Japan). The slides were stained with both HE and Ki67 to assess the morphology (data not shown). As positive controls during the staining, we used lymphoid tissue, as the protein has been described as highly expressed in tissue https://www.proteinatlas.org/ENSG00000103194-USP10/tissue, https://www.proteinatlas.org/ENSG00000135439-AGAP2/tissue.

### Survival analysis

Survival analyses were performed using the Kaplan–Meier log-rank and Wilcoxon signed-rank tests to evaluate progression-free survival (PFS) and overall survival, with events defined as progression or lack of progression. Endpoints were progression, death of the patient due to ccRCC, or PFS to the end of the follow-up period for this study (1.2.2020). Analyses were performed using R (v.1.1.383; R Foundation for Statistical Computing, Vienna, Austria; packages: Tidyverse and Survival). Hazard ratios were determined using JMP Genomics (Fit proportional hazards; SAS Institute), and survival curves were generated using SPSS (v.25; IBM Corp.).

### qPCR

qPCR was performed using SuperScript IV VILO master mix with ezDNase (No. 11766050; Thermo Fisher Scientific), TaqMan Fast Advanced master mix (No. 4444556; Thermo Fisher Scientific), and the *AGAP2-AS1 *primer and probe (Hs01096080_s1, no. 4426961; Thermo Fisher Scientific). qPCR was performed on a StepOne Plus real-time PCR system (Applied Biosystems, Carlsbad, CA, USA), with the gene encoding 40S ribosomal protein S13 (*RPS13;* Hs01011487_g1, no. 4426961; Thermo Fisher Scientific) used to normalize samples. RNA input for cDNA was 20 ng for serum and 50 ng for solid tissue. We used a no template control as negative control.

### Statistical analysis

mRNA abundance, qPCR analysis, and correlation plots were generated using SPSS (v.25; IBM Corp., Armonk, NY, USA), with correlations determined using Spearman’s rho test and continuous variables for age, creatinine level, *AGAP2-AS1* expression, tumor size, time to metastasis, and categorical variables for sex, Leibovich score, and sample status.

In qPCR, three technical replicates per sample were used to compile an average Ct value, which was used in subsequent analyses. qPCR analysis to determine abs. Fold change (FC) between groups was determined by averaging the normalized Ct values for each group and determining the ∆∆Ct with the averaged values. Significance and *p* values were evaluated using the Mann–Whitney *U *test according to the ∆Ct values from each sample.

Categorical variables, such as different nephrectomies, were analyzed with the Chi-squared Test.

#### Data availability

Data are available at the Gene Expression Omnibus (GEO) data repository (https://www.ncbi.nlm.nih.gov/geo/), GEO accession number GSE171955. External datasets from the Genomics Data Commons (GDC) and The Cancer Genome Atlas (TCGA) were accessed and analyzed via the UCSC-XENA website (https://xena.ucsc.edu).

## Results

### Patient selection

From the 443 ccRCC patient cohort, 274 were assigned a Leibovich score between zero and two (low-risk). Within this low-risk subgroup, 8 developed distant metastases within 109 days–7 years, with an average time to recurrence of 4 years and 8 months and were enrolled in this study as progressors. Two matched nonprogressors with similar Leibovich scores, Fuhrmann grades, tumor stages and sizes, similar creatinine levels, and similarly underwent surgical tumor removal but did not develop metastases in a 2–7 year time range (average follow-up: 6 years) per progressor sample were included as controls (n  = 16).

No statistically significant differences regarding age, creatinine levels, tumor size, tumor stage, Fuhrmann grade or Leibovich scores were found between the progressor and nonprogressor groups. However, 7/8 progressor patients were treated with radical nephrectomy compared to 9/16 nonprogressor patients (p  = 0.04) (Table 1). Follow-up details have been reported previously [15, 16].

### Differentially expressed genes (DEG)

**Fig. 1:**
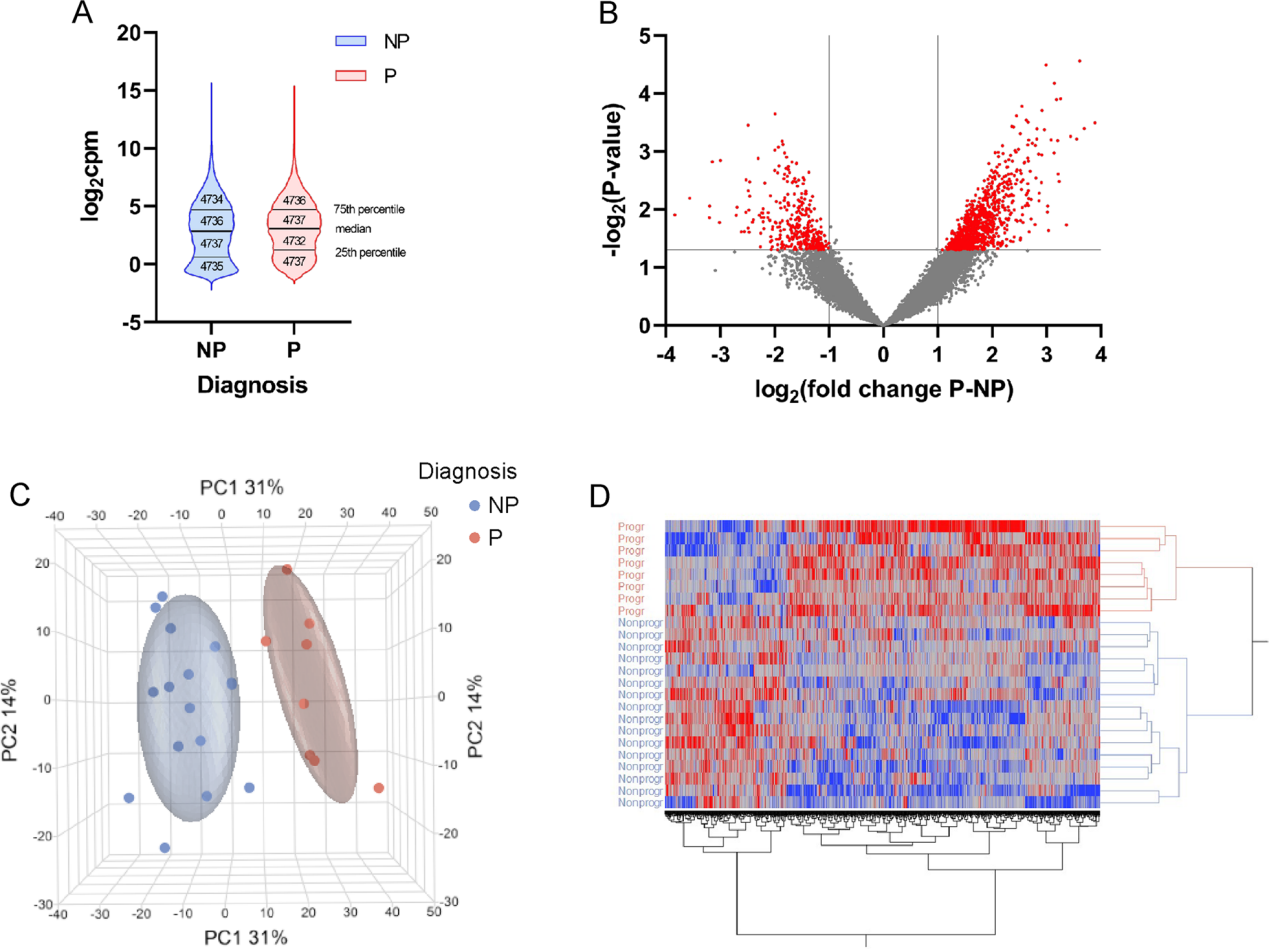
Data visualization. Comparison of violin plots of the distribution of the mean normalized expression values of 18,942 quality filtered ENSEMBL genes shows very similar distribution patterns for both patient groups (**A**). The median values were very similar for both groups (NP: 2.841, P: 3.080), with means being almost identical to the median values (NP: 2.835, P: 3.082). The volcano plot (**B**) of all 18,942 genes reveals a fairly symmetrical distribution of the fold changes in the absence of overt severe anomalies. Dots in red are 1167 differentially expressed genes. (**C**) Displays principal component analysis using expression values. A total of 1167 genes were differentially expressed (abs. FC  ≥ 2; *p * ≤ 0.05) in the nonprogressor (blue) and progressor (red) groups. Each dot represents one patient. The nonoverlapping circle surrounding each group refers to the 95% confidence interval. The nonprogressors and progressors cluster together and separate along PC1, which explains 31% of the variance. The heatmap in **D** is based on the same 1167 differentially expressed genes used in the PCA. All samples clustered within their own group, with a clear separation between progressors and nonprogressors. Upregulated genes are shown in shades of red, and downregulated genes are shown in shades of blue

A total of 18,942 genes were detected by applying an empirical filter that left genes with at least 1 cpm in 3 or more samples. Visual inspection of the groupwise mean of the genes indicated that the value distribution for both groups was very similar, with both the mean and median values almost identical (Fig. 1A). The symmetry of the volcano plot suggests that there is no bias toward overrepresentation of genes in a given group (Fig. 1B). A total of 1167 out of 18,942 detected genes were found to be differentially expressed between the groups “progressors” (P) and “nonprogressors” (NP). A total of 840 genes were upregulated and 327 were downregulated in tumors from progressors compared to those from nonprogressors. The top 20 most overrepresented genes according to FC are shown in Table 2. On average, the most overrepresented gene in nonprogressor samples compared to progressor samples was solute carrier family 12 member 1 (*SLC12A1*) gene FC  = 14,31; *p*  = 1.24e−2), whereas the most overrepresented gene in the progressor group compared to the nonprogressor group was the synaptic vesicle glycoprotein 2B gene (*SV2B*) (FC  = 14.84; *p*  = 3.19e−4).

**Table 2:** Top 20 upregulated and downregulated genes

Symbol	Ratio NP/P	Abs. FC	*P* Value
Most overrepresented in Nonprogr			
SLC12A1	14.31	14.31	1.24E−02
AC146944.1	11.82	11.82	6.37E−03
IGHA2	9.18	9.18	1.40E−02
IGLL5	9.13	9.13	8.78E−03
SLC9A4	8.84	8.84	1.51E−03
HPD	8.05	8.05	1.68E−02
IGKV1-5	7.98	7.98	1.44E−03
GRIA4	6.53	6.53	1.27E−02
H3F3AP4	6.46	6.46	9.16E−03
IGHV4-61	6.14	6.14	2.43E−02
SNORD115-11	5.89	5.89	2.41E−02
IGLV2-23	5.78	5.78	1.12E−02
IGLV2-14	5.77	5.77	3.10E−03
CLEC18C	5.62	5.62	2.11E−02
SNORA38	5.62	5.62	3.51E−04
MST1P2	5.60	5.60	4.75E−03
IGKV1-39	5.55	5.55	1.03E−02
MST1L	5.54	5.54	2.48E−02
LTF	5.50	5.50	3.37E−03
SYT7	5.5	5.50	0.00595
Most overrepresented in Progr			
SV2B	0.06737229	14.8429	0.000319
FAM86B2	0.077412071	12.91788	0.000404
NPIPB9	0.082203939	12.16487	2.76E-05
ANKRD20A7P	0.085199087	11.73721	0.000611
LBP	0.092082598	10.85982	0.000551
SNORD116-18	0.097088878	10.29984	0.01859
ADGRB1	0.10462215	9.558205	0.000124
ASAH2	0.106344157	9.403432	0.003289
PLK4	0.108067443	9.253481	0.002456
STAG3	0.108558859	9.211593	0.000654
ZNF321P	0.109969942	9.093394	0.000128
AC135048.4	0.113068345	8.844208	6.67E-05
NPIPB1P	0.113446458	8.814731	0.001067
SYS1-DBNDD2	0.118191167	8.460869	0.00063
RMRP	0.122271935	8.178492	0.023045
RASA4CP	0.123307134	8.109831	0.008782
AC239799.1	0.123616908	8.089508	0.011112
DCUN1D2	0.125942208	7.94015	3.24E-05
FAM131C	0.128131248	7.804497	0.000418
CAMK2B	0.132235909	7.562242	0.000196

Top 20 upregulated and downregulated genes in nonprogressor and progressor patients. The 20 genes with the lowest and highest FC are displayed. *AGAP-AS1* was not found among these genes

*Nonprogr* nonprogressors; *Progr* progressors

### Principal component analysis (PCA)

PCA was performed using normalized mRNA read counts of the 1167 differentially expressed genes (Fig. 1C). The two groups separated along PC1, which explained 31% of the variation. Furthermore, hierarchical clustering analysis using the 1167 differentially expressed genes also separated progressors from nonprogressors, with no patient clustering outside their respective group (Fig. 1D).

### Pathway analysis and gene set enrichment analysis

Pathway analysis of the 1167 differentially expressed gene sets revealed “Primary Immunodeficiency Signaling” (*p * = 6.76e−04) as the most represented canonical pathway, followed by “Molecular Mechanisms of Cancer” (*p*  = 1.28e−3). The top disease category was “Cancer”, with 169 “Disease function” categories and *p* values ranging from 7.49e−15 (“Nonmelanoma solid tumor”: 777 genes) to 1.47e−02 (“Laryngeal cancer”: 40 genes) (Additional file 2).

Gene set enrichment analysis (Table 3) did not yield many KEGG pathways that would have been enriched in either of the patient groups with statistical significance. This was not unexpected since we had very similar patient conditions in our analysis, with all patients suffering from mild stages of ccRCC at the time of biopsy. Enriched in NP was glycosaminoglycan degradation, and enriched in P was “Basel transcription factors”, “Hedgehog signaling pathway”, and “sphingolipid metabolism”.

**Table 3:** Gene set enrichment analysis

Gene sets	Gene set size	Normalized enrichment score	*P* value
KEGG_BASAL_TRANSCRIPTION_FACTORS	34	− 1.63	0.014
KEGG_HEDGEHOG_SIGNALING_PATHWAY	40	− 1.53	0.032
KEGG_SPHINGOLIPID_METABOLISM	36	− 1.46	0.043

Results from the gene set enrichment analysis in the progressor patients. Only results with a *p* value below 0.05 are displayed. Three pathways were significantly affected

### Identification of AGAP2-AS1 as prognostic marker

Unsupervised gene filtering analysis using the *k*-nearest neighbor was then used to evaluate the ability of all 1167 differentially expressed genes to correctly classify the 24 samples. We tested gene sets consisting of between one and 10 features and selected for their ability to predict disease progression (Additional file 3). The results were ranked for their ability to correctly classify the largest number of samples as either progressor or non-progressor.

**Fig. 2:**
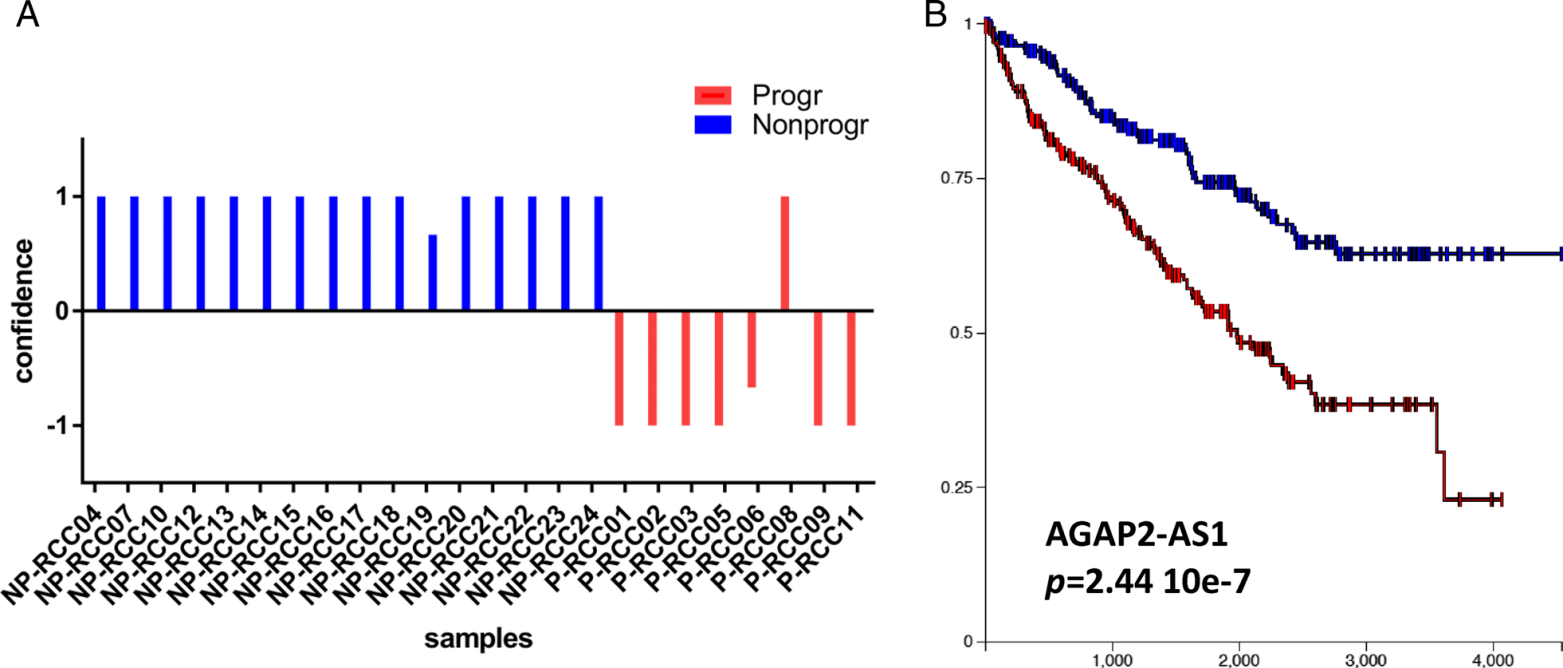
AGAP2-AS1 expression levels can be used to classify patients into those with poor and good prognoses. In **a**, the classification of n  = 8 progressors and n  = 16 matched nonprogressors is displayed. Each pillar represents one sample. Columns with negative confidence (y-axis) were classified as progressors, whereas positive values were classified as nonprogressors. Colors indicate the actual status as either progressor (red) or nonprogressor (blue). Correct classification was observed in 23 of 24 samples (sensitivity: 87.5%; specificity: 100%, area under the curve score 0.93). **b** Shows the results from mining the GDC TCGA ccRCC data on the prognostic significance of *AGAP2-AS1* for patients with ccRCC or controls. The blue lines represent patients with low expression of AGAP2-AS1, while the red lines represent high expression. Time is displayed on the x-axis, while the y-axis displays the percentage of surviving patients. Higher expression of AGAP2-AS1 is significantly associated with poor prognosis

The best results were achieved by using AGAP2 antisense RNA 1 (*AGAP2-AS1, p*  < 0.001, FC  = 5.82). *AGAP2-AS1* is an antisense RNA belonging to the family of long noncoding (lnc) RNAs. *AGAP2-AS1* gene expression alone was sufficient to correctly classify 23 of 24 samples (96%), thereby eliminating the need for a larger gene panel (Fig. 2a)**.** The only sample that was incorrectly classified was a progressor sample with a very low RNA yield (9.95 ng/sample), but the sample was not otherwise distinct. Neither did it systematically differ from the other samples and the results do not have a lower quality. No significant correlations between *AGAP2-AS1* expression level and tumor size, Leibovich score, patient age, sex, or creatinine level were observed. In a survival analysis where patients were split based on *AGAP2-AS1 expression*, low AGAP2-AS1 suggested good prognosis, and high levels indicated poor prognosis at a cutoff of 1.5 log_2_cpm normalized expression level.

Importantly, mining of existing databases (ccRCC GDC TCGA, https://portal.gdc.cancer.gov/projects/TCGA-KIRC) indicates that, irrespective of prognostic predictors, overexpression of *AGAP2-AS1* is associated with overall unfavorable survival outcome in ccRCC (Fig. 2b), thus providing corroboration of our findings.

To further validate the RNA-seq results, we performed RT–qPCR analysis of *AGAP2-AS1* gene expression in primary tumor tissue from a subset of the same samples used for RNAseq from progressors (n  = 7) and nonprogressors (n  = 7). In accordance with the RNAseq data, the *AGAP2-AS1* gene was overexpressed in progressors [p  = 0.035, FC (P/NP): 4.09]. Intriguingly, in our cohort, *AGAP2-AS1* gene expression did not differ between tumor biopsies from the original tumor and the metastasis in the same progressor patient (data not shown).

### Circulating AGAP-AS1 RNA

Analysis of serum *AGAP2-AS1* RNA levels in these samples provided initial evidence of overexpression of this gene in progressors. However, it failed to reach significance (p  = 0.12).

### Immunohistochemical analysis

As *AGAP2-AS1* is a long noncoding RNA, we explored the expression of the protein encoded by the complementary strand to *AGAP2-AS1*, ADP-ribosylation factor GTPase-activating protein with GTPase domain, ankyrin repeat, and PH domain 2 (AGAP2).

We observed a stronger AGAP2 signal in the progressor group (Additional file 4).

We also stained specimens for ubiquitin-specific peptidase 10 (USP10) protein [22] to examine whether transcriptomic results matched results at the protein level. The experiments revealed data in accordance with the RNAseq (FC NP/P: 3.20, p  < 0.01) (Additional file 4).

### Survival analysis

In keeping with selection criteria, i.e., progressors vs. nonprogressors, progression-free survival was confirmed to be significantly higher in the nonprogressor patients [*p * < 0.0005; hazard ratio (HR): 9.24e−11]. However, due to unrelated cancer-related death in three patients in the nonprogressor group, only a trend (p  = 0.07) toward shorter overall survival was observed in the progressor group (Additional file 5).

## Discussion

In this study, we investigated the gene expression profiles of ccRCC patients with an originally estimated low risk of progression who nevertheless developed metastasis during a follow-up period of up to eleven years.

Our main finding is that the level of *AGAP2-AS1* expression in tumor tissues from the time of the initial surgery correctly predicts 100% of the nonprogressor group and close to 90% of the progressor group of patients with low-risk ccRCC. Higher expression of *AGAP2-AS1* long noncoding RNA in progressors than in nonprogressors was further confirmed by qPCR.

Taken together, these data provide novel tools, contributing to a more effective prognostic profiling of patients with low-risk ccRCC.

Considering the low percentage of low-risk ccRCC patients developing disease progression [8, 12], it is reasonable to question whether they represent a transcriptomically distinct cohort. Indeed, PCA and hierarchical clustering results indicate that progressors and nonprogressors form distinct groups at the transcriptome level, and the identification of prospective biomarkers using qPCR is of potentially high clinical relevance.

Interestingly, qPCR analysis of serum samples also suggests a higher level of *AGAP2-AS1* circulating RNA in progressors, although the investigated cohort was too small to detect significant differences. If confirmed, these data would support the use of liquid biopsies compared to solid tissue samples in diagnosis [23]. As the liquid biopsies were taken at the time of surgery, we hypothesized that liquid biopsies taken later, but still prior to recurrence, may have contained higher levels of *AGAP2-AS1* in the progressor patients.

Conventional prognostic models, e.g., the Leibovich score, are characterized by high sensitivity and specificity to predict recurrence in ccRCC [10]. Furthermore, they are well established and do not require the use of additional sequencing techniques [9, 10, 12, 24].

However, *AGAP2-AS1* overexpression specifically detected metastasizing tumors classified as “low-risk” by the current methods. Combining conventional prognostic models and *AGAP2-AS1,* easily measured by PCR, would allow for a more accurate estimation of the risk profile of low-risk patients.

Once low-risk progressors have been identified, stratification could also allow for a reduced follow-up for low-risk nonprogressors, freeing valuable resources.

*AGAP2-AS1* is a long noncoding antisense RNA previously investigated in a variety of cancers [25–32], where its upregulation was shown to correlate with decreased survival rates [28, 33]. Accordingly, *AGAP2-AS1* silencing suppresses the proliferation and invasion potential of glioblastoma cells while promoting their apoptosis [34]. Multiple studies also show that *AGAP2-AS1* knockdown inhibits the proliferation of malignant cells from pancreatic [27] and hepatic cancers and gliomas [35, 36] in vitro and in vivo. Moreover, breast cancer cell lines overexpressing *AGAP2-AS1* and showing resistance to trastuzumab were resensitized to its effects following gene knockdown [26]. Interestingly, in a study comparing metastatic to localized prostate cancer, the *AGAP2-AS1* gene was also found to be upregulated in metastatic cancer tissues [37]. In ccRCC, using a cohort of n  = 611 samples *AGAP2-AS1* was found to significantly correlate with higher tumor stages, prognosis and metastasis in the TCGA dataset, corroborating our independent findings [38].

In our data, *AGAP2-AS1* was not differentially expressed between the original tumor and metastases. This could be taken to indicate stable gene expression over time, as the biopsy from the metastasis was taken an average of 4.5 years after the original biopsy.

A well-known transcriptomic characterization of ccRCC is represented by the classification into ccA and ccB subtypes [39–41]. The ccB subtype displays markedly improved disease-specific survival compared with ccA [40]. The ClearCode34 risk predictor was developed to forecast the ccA or ccB and prognostic group classification [42]. According to our data, of the 34 mRNAs used in ClearCode34, only receptor tyrosine kinase-like orphan receptor 2 (upregulated in progressors; abs. FC: 3.81; *p*  < 0.05) was differentially expressed. This lack of overlap might be explained by the small size of our cohort. However, it is also of note that the ccA and ccB subtypes developed within a more heterogeneous cohort and were not restricted to stage 1 tumors.

In the only other closely related report, Parasramka et al. [12] also investigated low-risk ccRCC progressors using RNA-seq. However, although their findings were supported by a validation cohort, they did not attempt validation with other laboratory techniques. Of the 10 differentially expressed genes identified in both their discovery and validation sets, only ASPM (abnormal spindle-like microcephaly associated protein) (abs. FC: 4.84; p  < 0.05) was also differentially expressed in our study. Of the 20 most upregulated genes in both progressors and 20 most upregulated genes in nonprogressors, none were found in the differentially expressed genes listed by the authors.

*AGAP2-AS1* was not included within their published results, but as data from this study are not publicly available, we could not elucidate whether *AGAP2-AS1* was not significant, nondetected and/or not identified. A possible explanation for why *AGAP2-AS1* was not found might lie in their more stringent requirements for significance. They set their cutoff for significance at *p*  < 0.001 and filtered out any genes with fewer than three patients expressing at 2 counts per million. While we used the more usual cut off at *p*  < 0.05 and filtered at 1 counts per million in at least three samples. As such they found 92 genes which were differentially expressed in their cohort, compared our 1167. Considering that multiple other studies, including TCGA data show that AGAP2-AS1 correlates with both survival, stage, progression and progression in specific stages [38], AGAP2-AS1 might well have been excluded by these requirements.

One of the only pathways enriched progressors in the GSEA results was ‘’SPHINGOLIPID METABOLISM’’. In a recent paper, several genes from that pathway were linked to ccRCC progression [43]. In our data, 5 of those 6 genes fit with the authors’ findings, i.e., the gene direction of the deregulation indicated a worse prognosis in the progressive patients.

Our study has several limitations. Only 8 patients out of the initial 443 (1.8%) were progressors within low-risk ccRCC, mainly due to the low frequency of this subtype. Furthermore, the adjusted *p* values were not significant for all mRNAs due to the comparison of intrinsically similar samples, e.g., two forms of histologically identical cancer types from closely matched patients. While a larger cohort is unlikely to overcome the later restriction [12], the former can and should be corrected in a larger validation cohort.

Another limitation is the follow-up time of the controls. Not all nonprogressors had a follow-up time longer than the longest time until metastasis in the progressor group. However, all pairs of progressors and matched nonprogressors included at least one nonprogressor with a longer follow-up time than the time to metastasis in the matched progressor. Still, we cannot categorically exclude that some of the nonprogressors will turn into progressors, albeit delayed compared to the original group. However, even if this were to occur, we have demonstrated that the groups differ transcriptomically and delayed progression would still be a prognostic model of significant value.

There is also the lack of 100% sensitivity in only using AGAP2-AS1. While the results for using AGAP2-AS1 on its own were good, we could have achieved 100% sensitivity by extended the classifier. We could have added more RNAs which identified the one progressor which was incorrectly classified by AGAP2-AS1, increasing sensitivity, potentially at the cost of accuracy. The decreased accuracy due to an increase in misclassified samples might have been worthwhile, as a high sensitivity allows for the ruling out of disease [44]. Interestingly, the sensitivity of the 10 classifier models with one to 10 gene members, always stayed at 87.5% (always one Prog sample falsely classified, though not always the same), while the specificity decreased slightly from 100 to 81.25% (in the model with 2 genes), and then to 87% (with three to 8 genes), and then back to 93.75% with 9 or ten genes. Therefore, the number of genes needed to achieve 100% sensitivity, the small sample size and only a single misclassification led us to conclude that the results are best presented a single component classifier. However, a single gene might well be insufficient to act as a classifier in a heterogenous cohort such as ccRCC. A clinical classifier would most likely require the inclusion of additional components in order to be viable, however in our results the best classifier was AGAP2-AS1 on its own.

## Conclusion

RNA-seq reveals different transcriptomic profiles in “low-risk” ccRCC progressors and nonprogressors. Our findings suggest that *AGAP2-AS1* might represent a clinically effective biomarker of subsequent tumor progression in conventionally staged low-risk tumors. These findings, however, require further validation.

## Supplementary Information

**Additional file 1: **Patient selection procedure. Out of 443 patients operated on for ccRCC between 1997 and 2014, 274 were classified as “low risk” (score 0–2, according to the Leibovich 2003 classification). Eight of them (2.9%) developed metastases. Control patients (n = 16) matched for age, primary tumor stage and size, Fuhrman grade and eGFR were selected from the 266 nonprogressing “low risk” patients. A detailed analysis of the clinicopathological characteristics of progressing and nonprogressing control patients is reported in Table 1 of the main text.

**Additional file 2: **Ingenuity pathway analyses for progressors vs. nonprogressors. The top results are presented in table format (**a**), and the top 10 canonical pathways are additionally presented as a graph (**b**). The top disease was “Cancer”, even though we compared two forms of cancer that were histologically identically and came from closely matched patients.

**Additional file 3: **Biomarker evaluation. Evaluation of the expression of individual genes or combinations of up to 10 genes as biomarkers. The best results were achieved with *AGAP2-AS1*, which correctly classified 23 out of 24 samples without the need for a larger gene panel.

**Additional file 4: **IHC, survival and mRNA abundance plots for USP10 and AGAP2. IHC, survival and mRNA abundance plots for USP10 and AGAP2, IHC analysis of USP10 and mRNA abundance plots revealed upregulated protein and gene expression levels in nonprogressors (**a**) compared to progressors (**b**). Images were taken from matched sets of samples and viewed at 40x magnification. **c** depicts the corresponding gene abundance plot, which is both in accordance with the protein expression data and statistically significant (p = 0.0015). IHC analysis of AGAP2 and mRNA abundance plots for complementary *AGAP2-AS1 *(**f**) revealed upregulated protein and gene expression levels in progressors (**e**) compared to nonprogressors (**d**).

**Additional file 5: **Overall survival of patients. Overall survival of patients bearing nonprogressors (blue) and progressors (red) ccRCC (p = 0.078). During the follow-up, three deaths unrelated to ccRCC diagnosis also occurred in the nonprogressor group. All other patients were censored once the end of their follow-up was met.

## Data Availability

The datasets generated and analyzed during the current study are available in the Gene Expression Omnibus (GEO) data repository (https://www.ncbi.nlm.nih.gov/geo/), GEO Accession Number GSE171955.

## Abbreviations

ccRCC: Clear cell renal cell carcinoma
FFPE: Formalin-fixed and paraffin-embedded
IHC: Immunohistochemistry
PFS: Progression-free survival
PCA: Principal component analysis
abs.FC: Absolute fold change
FC: Fold change
Lnc: Long noncoding
