# Supplementary material for: AGAP2-AS1 as a prognostic biomarker in low-risk clear cell renal cell carcinoma patients with progressing disease
Source: Cancer Cell Int. 2021 Dec 20;21:690. doi: 10.1186/s12935-021-02395-9 (PMC8686242; doi:10.1186/s12935-021-02395-9)
Supplement: Supplementary file 1 — Additional file 1: Patient selection procedure. Out of 443 patients operated on for ccRCC between 1997 and 2014, 274 were classified as “low risk” (score 0–2, according to the Leibovich 2003 classification). Eight of them (2.9%) developed metastases. Control patients (n = 16) matched for age, primary tumor stage and size, Fuhrman grade and eGFR were selected from the 266 nonprogressing “low risk” patients. A detailed analysis of the clinicopathological characteristics of progressing and nonprogressing control patients is reported in Table 1 of the main text. [file 12935_2021_2395_MOESM1_ESM.pdf]

Additional file 1: Patient selection procedure

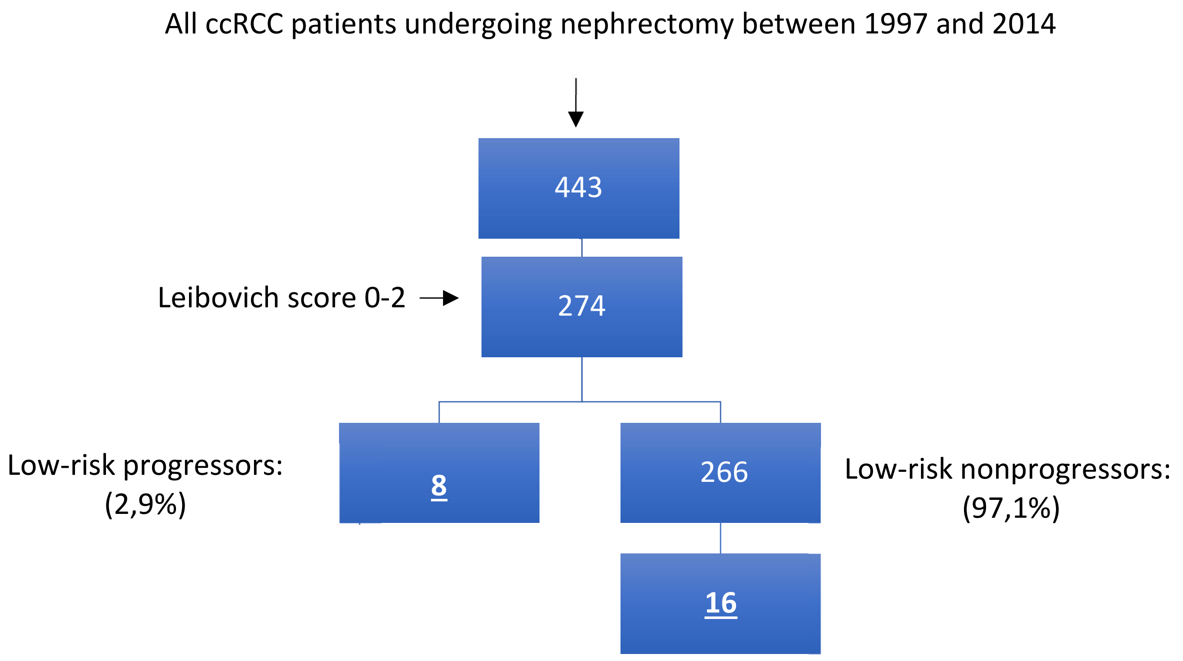

Additional file 1: Out of 443 patients operated for ccRCC between 1997 and 2014, 274 were classified as “low risk” (score 0-2, according to Leibovich 2003 classification). Eight of them (2.9%) developed metastases. Control patients (n=16) matched for age, primary tumor stage and size, Fuhrman grade and eGFR were selected from the 266 non-progressing “low risk” patients. A detailed analysis of the clinical-pathological characteristics of progressing and non-progressing control patients is reported in **Table 1** of the main text
