# Supplementary material for: AGAP2-AS1 as a prognostic biomarker in low-risk clear cell renal cell carcinoma patients with progressing disease
Source: Cancer Cell Int. 2021 Dec 20;21:690. doi: 10.1186/s12935-021-02395-9 (PMC8686242; doi:10.1186/s12935-021-02395-9)
Supplement: Supplementary file 2 — Additional file 2: Ingenuity pathway analyses for progressors vs. nonprogressors. The top results are presented in table format (a), and the top 10 canonical pathways are additionally presented as a graph (b). The top disease was “Cancer”, even though we compared two forms of cancer that were histologically identically and came from closely matched patients. [file 12935_2021_2395_MOESM2_ESM.pdf]

Additional file 2: Ingenuity Pathway analysis

2a: results from Top Canonical Pathways, Diseases and Disorders, Nephrotoxicity and Top Upstream Regulators.

Top Canonical Pathways	p-value	Overlap
Primary Immunodeficiency Signalling	6.76E-04	16.0% 8/50
Molecular Mechanisms of Cancer	1.28E-03	7.2 % 29/402
B Cell Receptor Signalling	2.02E-03	8.6 % 17/197
Triacylglycerol Biosynthesis	2.53E-03	14.6 % 7/48
Retinoate Biosynthesis I	9.94E-03	14.7 % 5/34

Diseases and Disorders	p-value range	# Molecules
Cancer	1.47E-02 – 7.49E-15	797
Organismal Injury and Abnormalities	1.47E-02 – 7.49E-15	801
Gastrointestinal Disease	1.46E-02 – 5.60E-09	692
Endocrine System Disorders	1.42E-02 – 1.16E-08	634
Dermatological Diseases and Conditions	1.42E-02 – 1.60E-07	470

Nephrotoxicity	p-value range	# Molecules
Renal Atrophy	5.69E-01 – 4.23E-03	3
Renal Inflammation	1.00E00 – 1.42E-02	14
Renal Nephritis	1.00E00 – 1.42E-02	14
Nephrosis	4.58E-01 – 2.30E-02	6
Renal Hydronephrosis	7.69E-02 – 3.49E-02	8

Top Upstream Regulators	p-value	Predicted Activation
CDK4	2.08E-05	
miR-145-5p (and other miRNAs w/seed UCCAGUU)	5.18E-04	
CCND1	5.79E-04	Inhibited
gefitinib	5.84E-04	
ITGA9	1.30E-03	

2b: Top 10 canonical pathways

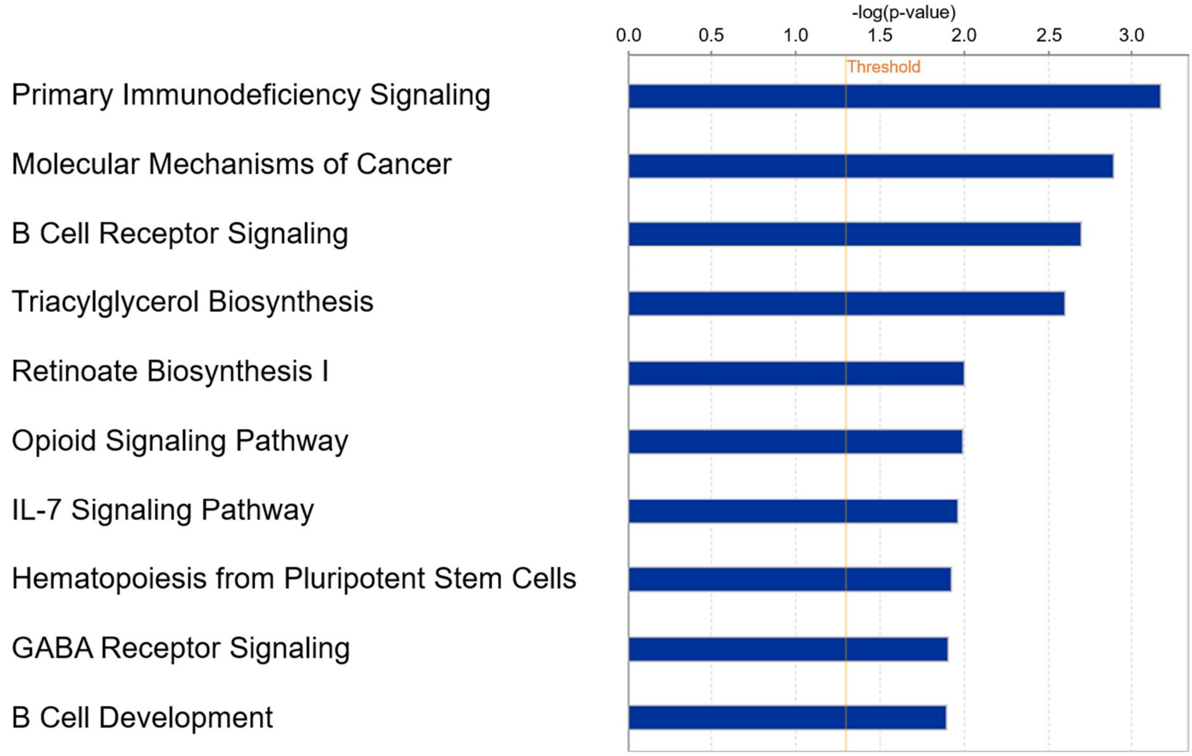

Additional file 2: Ingenuity Pathway Analyses for progressors VS non-progressors. The top results are presented in table format (**2a**) and the Top 10 Canonical Pathways are additionally presented as a graph (**2b**). The top disease was “Cancer”, even though we compared two forms of cancer that were histologically identically and came from closely matched patients.
