# Supplementary material for: AGAP2-AS1 as a prognostic biomarker in low-risk clear cell renal cell carcinoma patients with progressing disease
Source: Cancer Cell Int. 2021 Dec 20;21:690. doi: 10.1186/s12935-021-02395-9 (PMC8686242; doi:10.1186/s12935-021-02395-9)
Supplement: Supplementary file 4 — Additional file 4: IHC, survival and mRNA abundance plots for USP10 and AGAP2. IHC, survival and mRNA abundance plots for USP10 and AGAP2, IHC analysis of USP10 and mRNA abundance plots revealed upregulated protein and gene expression levels in nonprogressors (a) compared to progressors (b). Images were taken from matched sets of samples and viewed at 40x magnification. c depicts the corresponding gene abundance plot, which is both in accordance with the protein expression data and statistically significant (p = 0.0015). IHC analysis of AGAP2 and mRNA abundance plots for complementary AGAP2-AS1 (f) revealed upregulated protein and gene expression levels in progressors (e) compared to nonprogressors (d). [file 12935_2021_2395_MOESM4_ESM.pdf]

Additional file 4: IHC, Survival and mRNA-abundance plots for USP10 and AGAP2.

4a non-Progressor **4b** Progressor **4c**

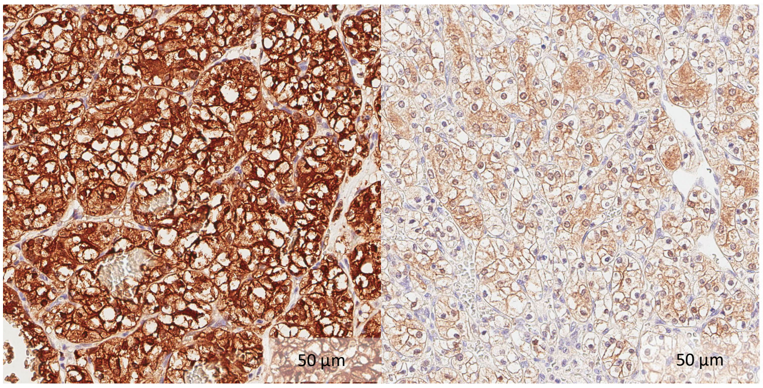

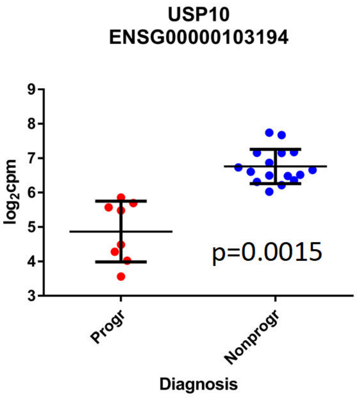

4d **4e** **4f**

non-progressor Progressor

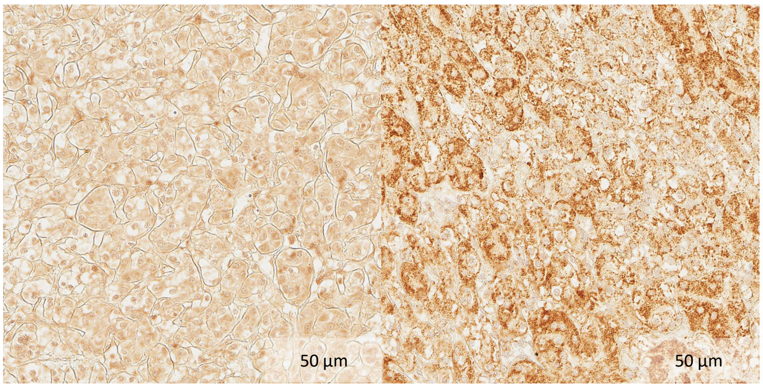

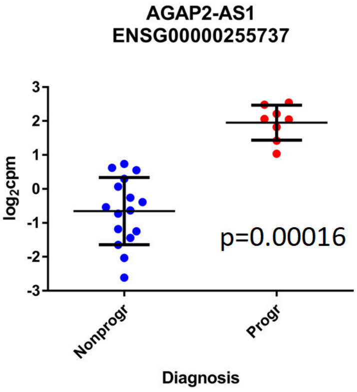

Additional file 4: IHC, Survival and mRNA-abundance plots for USP10 and AGAP2. IHC analysis of USP10 and mRNA-abundance plots revealed upregulated protein and gene expression levels in non-progressors (**4a)** as compared to progressors (**4b)**. Images were taken from matched sets of samples, viewed at 40x magnification. **4c** depicts the corresponding gene abundance plot, which is both in accordance with the protein expression data and statistically significant (p=0.0015). IHC analysis of AGAP2 and mRNA-abundance plots for the complementary *AGAP2-AS1* **(4f)** revealed upregulated protein and gene expression levels in progressors **(4e)** as compared to non-progressors **(4d)**
