# Supplementary material for: AGAP2-AS1 as a prognostic biomarker in low-risk clear cell renal cell carcinoma patients with progressing disease
Source: Cancer Cell Int. 2021 Dec 20;21:690. doi: 10.1186/s12935-021-02395-9 (PMC8686242; doi:10.1186/s12935-021-02395-9)
Supplement: Supplementary file 5 — Additional file 5: Overall survival of patients. Overall survival of patients bearing nonprogressors (blue) and progressors (red) ccRCC (p = 0.078). During the follow-up, three deaths unrelated to ccRCC diagnosis also occurred in the nonprogressor group. All other patients were censored once the end of their follow-up was met. [file 12935_2021_2395_MOESM5_ESM.pdf]

Additional file 5: Overall survival of patients bearing non-progressors (blue) and progressors (red)

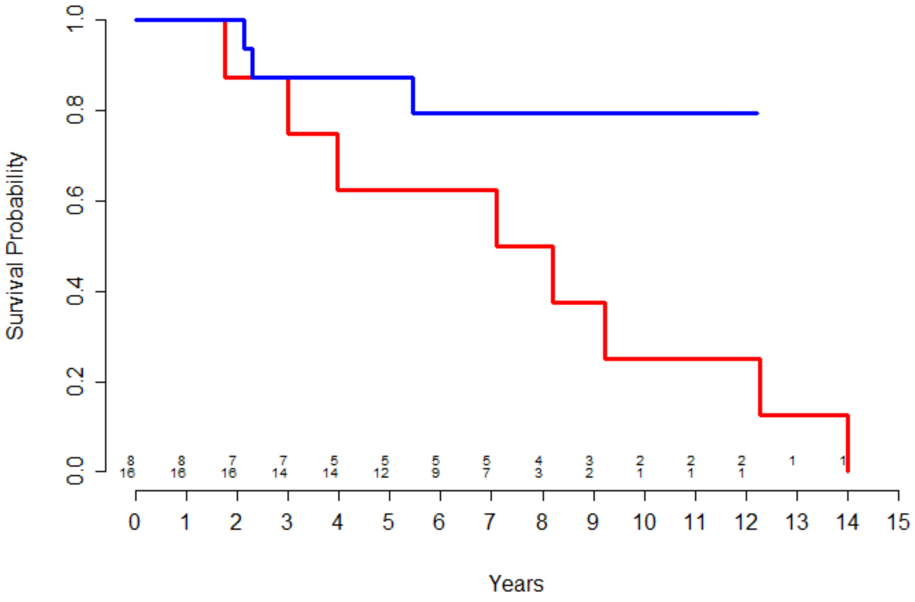

Additional file 5: Overall survival of patients bearing non-progressors (blue) and progressors (red) ccRCC (p=0.078). During the follow-up three deaths, unrelated to ccRCC diagnosis, also occurred in the non-progressor group. All other patients were censored once the end of their follow-up was met
